# Evaluation of arterial and venous ophthalmic hemodynamics in preeclamptic pregnant women

**DOI:** 10.1186/cc10923

**Published:** 2012-03-20

**Authors:** EM Shifman, NV Khramchenko, SV Sokologorskiy

**Affiliations:** 1 Federal Centre for Obstetrics, Gynecology & Neonatology, Moscow, Russia

## Introduction

The aim of the study was to evaluate arterial and venous ophthalmic blood flow parameters in mild and severe preeclampsia pregnancies and in normotensive pregnancies.

## Methods

A total of 117 women 25 to 30 years old with singleton pregnancies 30 to 40 weeks of gestation were recruited. Among them 40 pregnant women developed severe preeclampsia, 42 mild preeclampsia, and 35 were normotensive. Using color flow mapping (CFM) and pulse-wave Doppler imaging (PWD), maximum blood flow velocity (mFV) in the right/left arterial and venous ophthalmics along with Gosling's Doppler pulsatility index (PI) [1] in both arterial ophthalmics were evaluated. Mean blood pressure in all patients was also registered.

## Results

The highest mFV values (59.2 ± 4.61 and 23.6 ± 4.03 cm/second) were in the severe preeclampsia group while in the mild preeclampsia group mFV increased slightly or remained normal (35.6 ± 2.97 and 13.6 ± 0.81 cm/second). There was no mFV increase in the normotensive pregnancy group (31.5 ± 2.21 cm/second). No significant correlation was found between gestation age and mentioned hemodynamic parameters in the normotensive pregnancy group. PI values in the arterial ophthalmic in normotensive pregnant women were 2.92 ± 0.59 and the highest in all groups. In group with mild preeclampsia this parameter was 1.47 ± 0.30 and the lowest one was in patients with severe preeclampsia - 1.17 ± 0.08.

## Conclusion

In women with preeclampsia significant changes in ophthalmic hemodynamics take place - mFV in arterial and venous ophthalmics increases while PI values go down. This might be evidence of orbital hyperperfusion in preeclamptic pregnant women. Low PI values may be used as the markers of severe preeclampsia.
